# Call for morphological detection of Charcot‐Leyden crystals in tissue

**DOI:** 10.1002/clt2.12276

**Published:** 2023-07-03

**Authors:** Yurong Bai, Wenyi Chen, Weifeng Kong, Xin Luo, Jingyuan Chen, Xinyue Wang, Qingwu Wu, Jianning Chen, Qintai Yang, Yana Zhang

**Affiliations:** ^1^ Department of Otolaryngology‐Head and Neck Surgery The Third Affiliated Hospital of Sun Yat‐Sen University Guangzhou China; ^2^ Department of Pathology The Third Affiliated Hospital of Sun Yat‐Sen University Guangzhou China; ^3^ Department of Allergy The Third Affiliated Hospital of Sun Yat‐Sen University Guangzhou China

**Keywords:** CLCs, CRSwNP, ENT, immunology, morphology

To the editor,

We thank Dr. Sasaki, Dr. Suzaki, and Dr. Ueki for their interest and comments on the letter entitled “Predictive significance of Charcot‐Leyden crystal structures for nasal polyp recurrence”,^1^ which raises several important points. We agree with Ueki et al. that detecting Charcot‐Leyden crystals (CLCs) in tissue holds significant importance. Both data from us and others have demonstrated that CLCs serve as biomarkers for diagnosing eosinophilic chronic rhinosinusitis with nasal polyps (NPs) and predicting NP recurrence.^1^, ^2^ Although CLC protein has three distinct forms, including extracellular vesicles, extracellular soluble protein and crystalline CLC structures (CLCs), only crystalline CLCs could drive type 2 inflammation and allergic immunity.^3^, ^4^ Therefore, we think that detecting crystalline CLCs in tissue is crucial and meaningful. Despite both hematoxylin and eosin (H&E) staining and immunofluorescence (IF) histochemistry are methods for detecting crystalline CLCs, IF staining has long been supposed to have better efficiency and specificity in displaying crystalline CLCs morphology. Interestingly, contrary to our expectations based on the available literature, we found H&E staining has similar capability with IF staining for CLCs detection in NPs (Figure 1A,B). Moreover, counts of CLCs did not show any significant statistical difference between the two methods (Figure 1C). These results suggest that H&E staining may stand for a routine test for crystalline CLCs detection due to its accuracy and efficiency in clinical settings. Therefore, we advocate that pathologists should be encouraged to report the presence of CLCs in addition to eosinophils to clinicians in routine clinical practice, which will facilitate the diagnosis of NP endotypes, evaluate corticosteroid sensitivity, and even predict recurrence.

**FIGURE 1:**
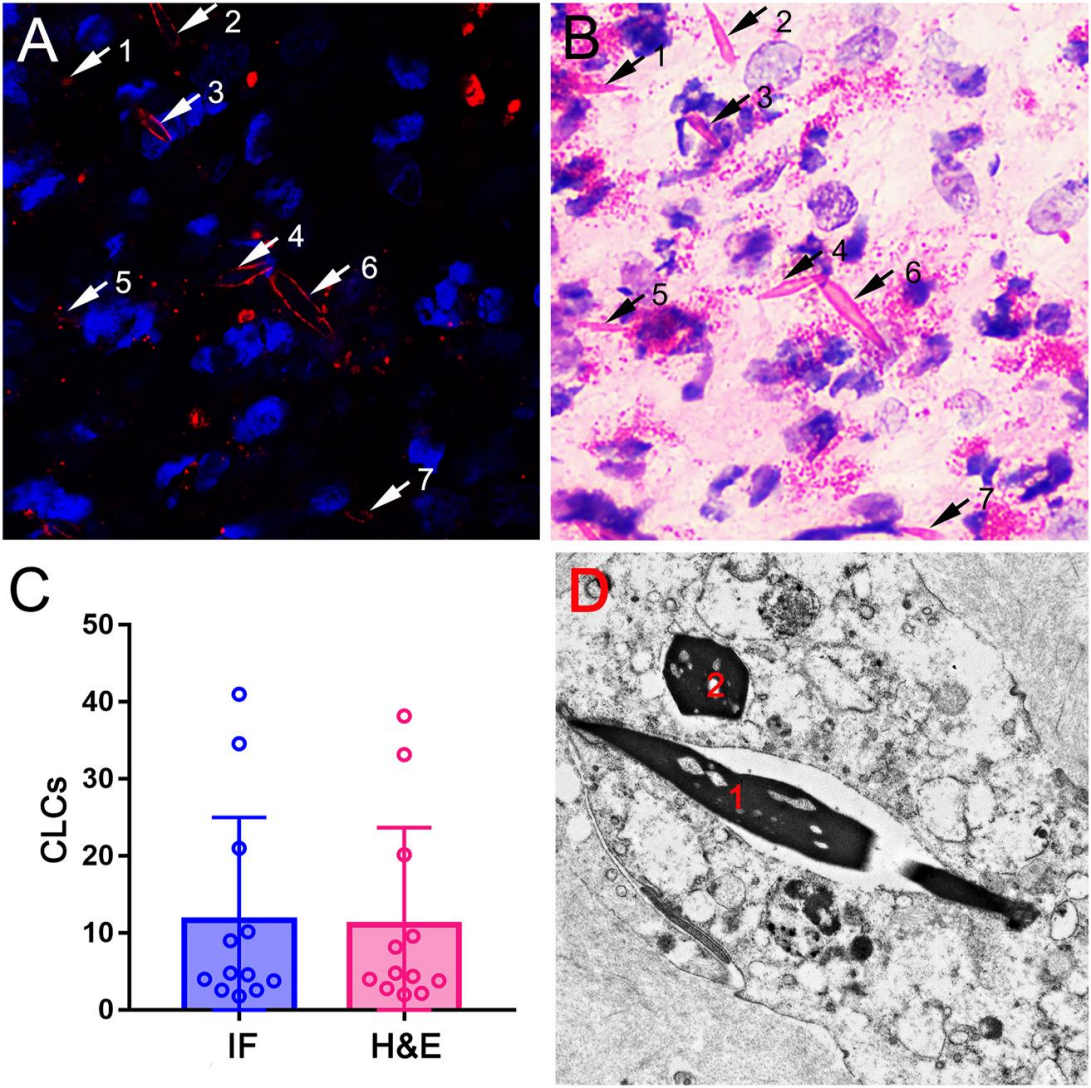
H&E staining has similar capability with IF staining for crystalline CLCs detection in NPs. The CLCs in the eosinophilic NP section were visualized using IF staining for primary mouse anti‐galectin‐10 mAb and Alexa‐555 conjugated goat anti‐mouse IgG to yield a red fluorescence product (A), followed by H&E counterstaining for the same visual field (B). Arrows with the same number indicate the same CLCs detected with IF (A) and H&E (B), respectively. Data plots for CLCs count detected using H&E staining and IF staining in eosinophilic NPs (C). Transmission electron microscopy showing the bipyramidal (D1) and hexagonal (D2) ultrastructure of CLCs in different angles (D). CLCs, Charcot‐Leyden crystals; NP, nasal polyp; IF, immunofluorescence; H&E, hematoxylin and eosin. The current study was approved by the Ethics Committee of the Third Affiliated Hospital of Sun Yat‐sen University (ID: II2023‐117‐01) and was conducted with written informed consent from patients.

However, we readily acknowledge that detection of CLCs by H&E staining has some issues as proposed by Ueki et al. First, we believe that the most critical factor determining staining success is the optimal eosin pH value. Actually, many commercial eosin solutions could detect eosinophils perfectly but not CLCs. It should also be noted that CLCs identified by H&E staining may be interfered by other eosinophilic components, which can be improved by optimizing the eosin solution pH value. Second, CLCs count may vary depending on the different fields of view. In fact, CLCs are preferentially detected in the nasal mucosa tissue that is in close proximity to denuded epithelium and eosinophil degranulation.^5^, ^6^ Artificial intelligence whole‐slide scanning counting is expected to be applied to reduce sampling errors in the future. Third, different angles of CLCs presented on tissue section may lead to the difficulty in identifying the characteristic bipyramid structure both by H&E and IF. Although transmission electron microscopy can identify the bipyramidal and hexagonal ultrastructures of CLCs (Figure 1D), it is not likely to be practical on a wide‐scale basis in routine clinical settings due to prohibitive cost constraints, complicated processing protocol, and limited observed field. Overall, we recommend that pathologists are encouraged to report CLCs count using H&E staining in clinical practice. Whether CLCs detection in tissue will help to further refine tailored therapy warrants further evaluation.

## AUTHOR CONTRIBUTIONS

**Yurong Bai**: Data curation (lead); Formal analysis (lead); Methodology (lead); Writing – original draft (lead). **Wenyi Chen**: Data curation (equal); Methodology (equal); Validation (equal). **Weifeng Kong**: Methodology (supporting); Software; (equal). **Xin Luo**: Methodology (supporting); Software (supporting). **Jingyuan Chen**: Methodology (supporting); Software (equal). **Xinyue Wang**: Methodology (supporting); Software (equal). **Qingwu Wu**: Methodology (supporting); Software (equal). **Jianning Chen**: Methodology (supporting); Resources (equal). **Qintai Yang**: Funding acquisition (supporting); Investigation (equal); Project administration (equal); writing – review & editing (equal). **Yana Zhang**: Conceptualization (lead); Funding acquisition (lead); Investigation (equal); Project administration (lead); Resources (lead); Supervision (lead); writing – original draft (supporting); writing – review and editing (lead).

## CONFLICT OF INTEREST STATEMENT

None.

## FUNDING INFORMATION

The National Natural Science Foundation of China (NSFC), Grant/Award Numbers: 8217114, U20A20399, 82271148; the Natural Science Foundation of Guangdong Province, Grant/Award Number: 2022A1515011787; the Science and Technology Program of Guangzhou, Grant/Award Number: 202201020402; the Key‐area Research and Development Program of Guangzhou Province, Grant/Award Number: 2020B0101130015; Fundamental Research Funds for the Central University, Sun Yat‐sen University, Grant/Award Number: 23qnpy141

## Data Availability

The data that support the findings of this study are available from the corresponding author upon reasonable request.
